# Immune-metabolic mechanisms of post-traumatic stress disorder and atherosclerosis

**DOI:** 10.3389/fphys.2023.1123692

**Published:** 2023-02-08

**Authors:** Yali Tian, Hanif Ullah, Jun Gu, Ka Li

**Affiliations:** ^1^ West China School of Nursing/West China Hospital, Sichuan University, Chengdu, China; ^2^ Department of Cardiovascular Surgery, West China Hospital, Sichuan University, Chengdu, China

**Keywords:** post-traumatic stress disorder, atherosclerosis-, metabolism, immune, AMPK/mTOR, PI3K/AKT

## Abstract

The interaction of post-traumatic stress disorder (PTSD) and atherosclerosis (AS) increase the risk of mortality. Metabolism and immunity play important roles in the comorbidity associated with PTSD and AS. The adenosine monophosphate-activated protein kinase/mammalian target of rapamycin and phosphatidylinositol 3-kinase/Akt pathways are attractive research topics in the fields of metabolism, immunity, and autophagy. They may be effective intervention targets in the prevention and treatment of PTSD comorbidity with AS. Herein, we comprehensively review metabolic factors, including glutamate and lipid alterations, in PTSD comorbidity with AS and discuss the possible implications in the pathophysiology of the diseases.

## 1 Introduction

Post-traumatic stress disorder (PTSD) is characterized by persistent maladaptive reactions after exposure to severe natural or psychological traumatic events. Traumatic events, including violent personal assaults, natural and anthropogenic disasters, and military combat or warfare, may trigger PTSD (O’Donnell et al., 2021). PTSD can be highly co-morbid with serious physical illnesses, including autoimmune diseases (Bookwalter et al., 2020), and cardiovascular diseases (CVD) (Wilson et al., 2019). Recent evidence demonstrates an association between PTSD and CVD along with major CVD outcomes such as coronary heart disease (CHD), myocardial infarction, and heart failure (Edmondson and von Knel, 2017). However, it is unclear whether these associations are causal or confounding. Furthermore, the biological and behavioral mechanisms underlying these associations are poorly understood. Some researchers have hypothesized that metabolic abnormalities and immune inflammatory responses play important roles in the comorbidity of PTSD and CHD (O’Donnell et al., 2021). Atherosclerosis (AS) is the main cause of CHD, cerebral infarction, and peripheral vascular lesions. In AS, affected artery lesions start from the intima, typically with deposition of lipids and complex sugars and thrombosis, followed by fibrous tissue hyperplasia, calcareous deposition, and gradual transformation and calcification of the middle layer of the artery. These alterations lead to thickening and hardening of the arterial wall and narrowing of the vascular lumen (Rossetti et al., 2015). Herein, we comprehensively review the metabolic factors, including glutamate and lipids, altered in PTSD comorbidity with AS and discuss the possible implications of the pathophysiology of the adenosine monophosphate (AMP)-activated protein kinase (AMPK)/mammalian target of rapamycin (mTOR) and phosphatidylinositol 3-kinase (PI3K)/protein kinase B (Akt) pathways through interactions with metabolism and autophagy.

## 2 Interaction between post-traumatic stress disorder and atherosclerosis

**FIGURE 1:**
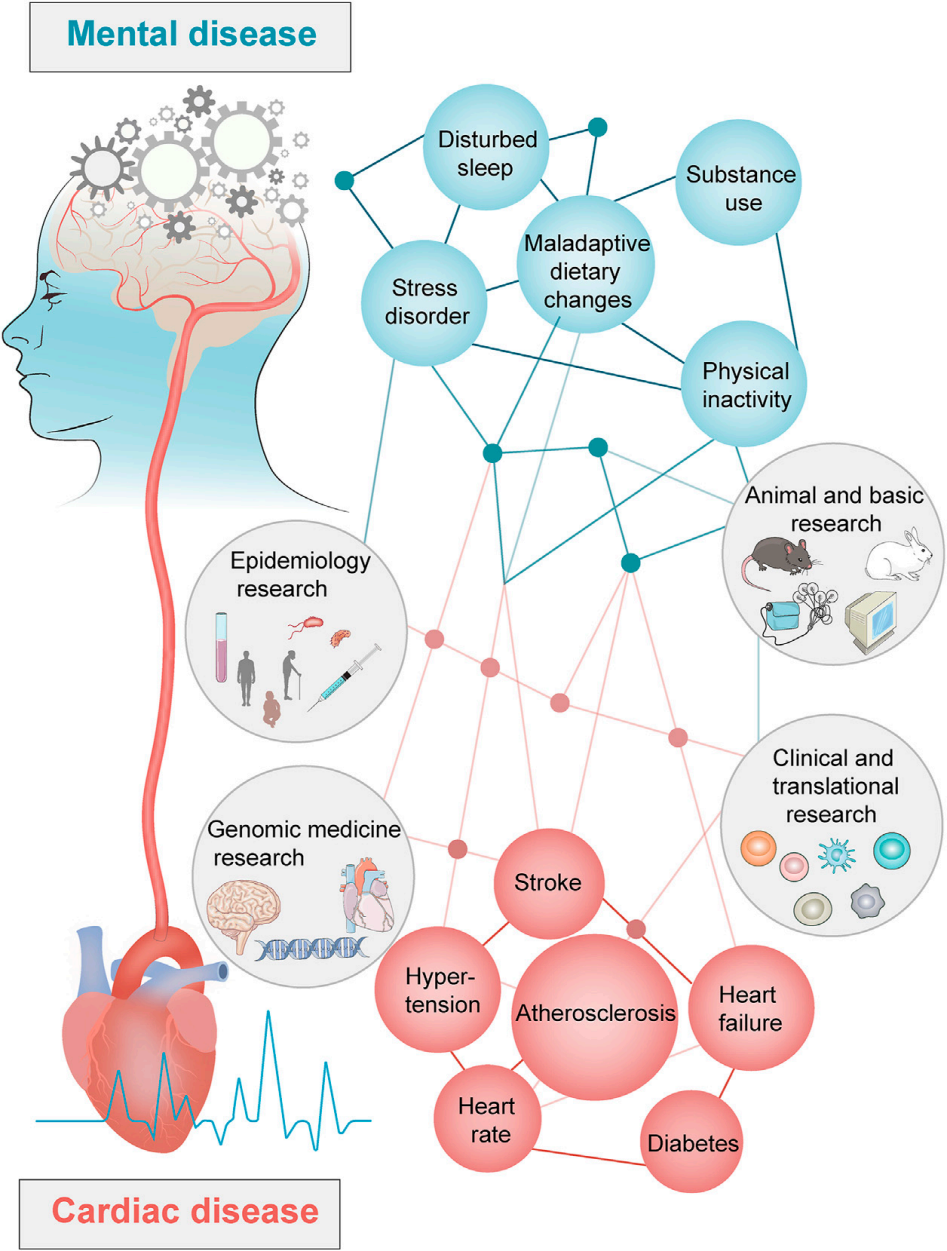
Interaction between mental diseases and cardiac diseases.

PTSD is a serious chronic emotional response to a traumatic event in which individuals exhibit severe environmental stress with symptoms of re-experience, avoidance, and hyper-arousal (Fossion et al., 2015). Following the COVID-19 outbreak, 6%–53.8% of people worldwide have developed PTSD symptoms due to stress (Phan et al., 2020). According to a study in 2021 (O’Donnell et al., 2021), mental stress causes pathophysiological changes in the central and peripheral nervous, immune, endocrine, and vascular systems. Studies published in the British Medical Journal, JAMA Cardiology, and Circulation demonstrate that PTSD increases the risk of CVD by 1–3 times and is also closely associated with an increased risk of cardiovascular events (such as myocardial infarction and stroke), progression of cardiovascular disease to heart failure, and premature death (Rossetti et al., 2015; Roy et al., 2015; Song et al., 2019; Phan et al., 2020; Ebrahimi et al., 2021). PTSD is an independent risk factor for CHD and increases the risk by 61% (Ebrahimi et al., 2021). PTSD also increases the risk of stroke caused by myocardial infarction by 2.37 times (Roy et al., 2015). The relationship between PTSD and CHD is mediated by specific genes, proteins, and metabolic pathways (Figure 1).

PTSD and CHD interact during the pathological process, thereby increasing the risk of death and affecting the outcome of patients (O’Donnell et al., 2021). An increasing number of studies have shown that the pathological process of AS is accompanied by an increase in anxiety-like behaviors and cognitive impairment. Moreover, AS is positively correlated with leukoencephalopathy and cognitive impairment (Ahmed et al., 2012; Rossetti et al., 2015; Roy et al., 2015; Song et al., 2019; Phan et al., 2020; Ebrahimi et al., 2021). As the medical model has changed from a simple biomedical to a bio-psycho-social medical model, increasing attention is being paid on the “two-heart” medical concept in the face of increasing mortality of patients with AS and PTSD comorbidity, advocating for increased focus on the high incidence of comorbidity by medical workers (O’Donnell et al., 2021).

## 3 Interaction of gut microbiome in post-traumatic stress disorder and atherosclerosis

Increasing evidence suggests a link between gut microbiota abnormalities and PTSD (Laudani et al., 2023). Neurological and neuropsychiatric disorders are associated with changes in the composition of the gut microbiota (Cryan et al., 2019). Stress-related conditions including major depressive disorder and PTSD are among the neuropsychiatric disorders that have been linked to alterations in the composition of the gut microbiota (Foster et al., 2017). Most of the studies in the field have been performed by using chronic stress models major depressive disorder, whereas little is known about the association between alteration in gut microbiota composition and acute traumatic stress-induced susceptibility/resilience mechanisms, which are more linked to PTSD. PTSD is a trauma- and stressor-related disorder that often occurs after exposure to a single traumatic event (Musazzi et al., 2018). The firstly supported by clinical studies showing altered gut microbiota composition in individuals with PTSD compared to trauma-exposed resilient individuals (Stanislawski et al., 2021; Malan-Muller et al., 2022). In particular, alterations of certain phyla (Actinobacteria, Lentisphaerae, Verrucomicrobia, and Olsenella), despite no significant changes in microbial alpha and beta diversity, were found to be correlated with clinician-administered posttraumatic stress disorder scale scores exhibited by people with PTSD (Hemmings et al., 2017; Malan-Muller et al., 2022). It has been also suggested that changes in gut microbiota composition may be directly linked to the dysregulation of the hypothalamic–pituitary–adrenal axis and glucocorticoid signaling characterizing individuals with PTSD (Foster et al., 2017). Recently, many studies have demonstrated that there are some relationships between microbiota and atherosclerosis. Atherosclerosis have been related to gut microbiota dysbiosis with an increase in the Firmicutes/Bacteroidetes ratio *via* productions of acetate and decreasing of butyrate. Butyrate, once proved to be the main energetic resource of intestinal epithelial cells (IECs), is able to maintain the stability of gut barrier. High-fat intake thought as the risk factor for atherosclerosis can induce remarkable changes in gut microbiota composition (Birchenough et al., 2019; Paone and Cani, 2020). Many researchers have also found that compared with people without atherosclerosis, the patients with atherosclerosis have differences in the gut microbiota (Karlsson et al., 2012; Ziganshina et al., 2016; Jie et al., 2017).

## 4 Effects of glutamate on post-traumatic stress disorder comorbidity with atherosclerosis

**FIGURE 2:**
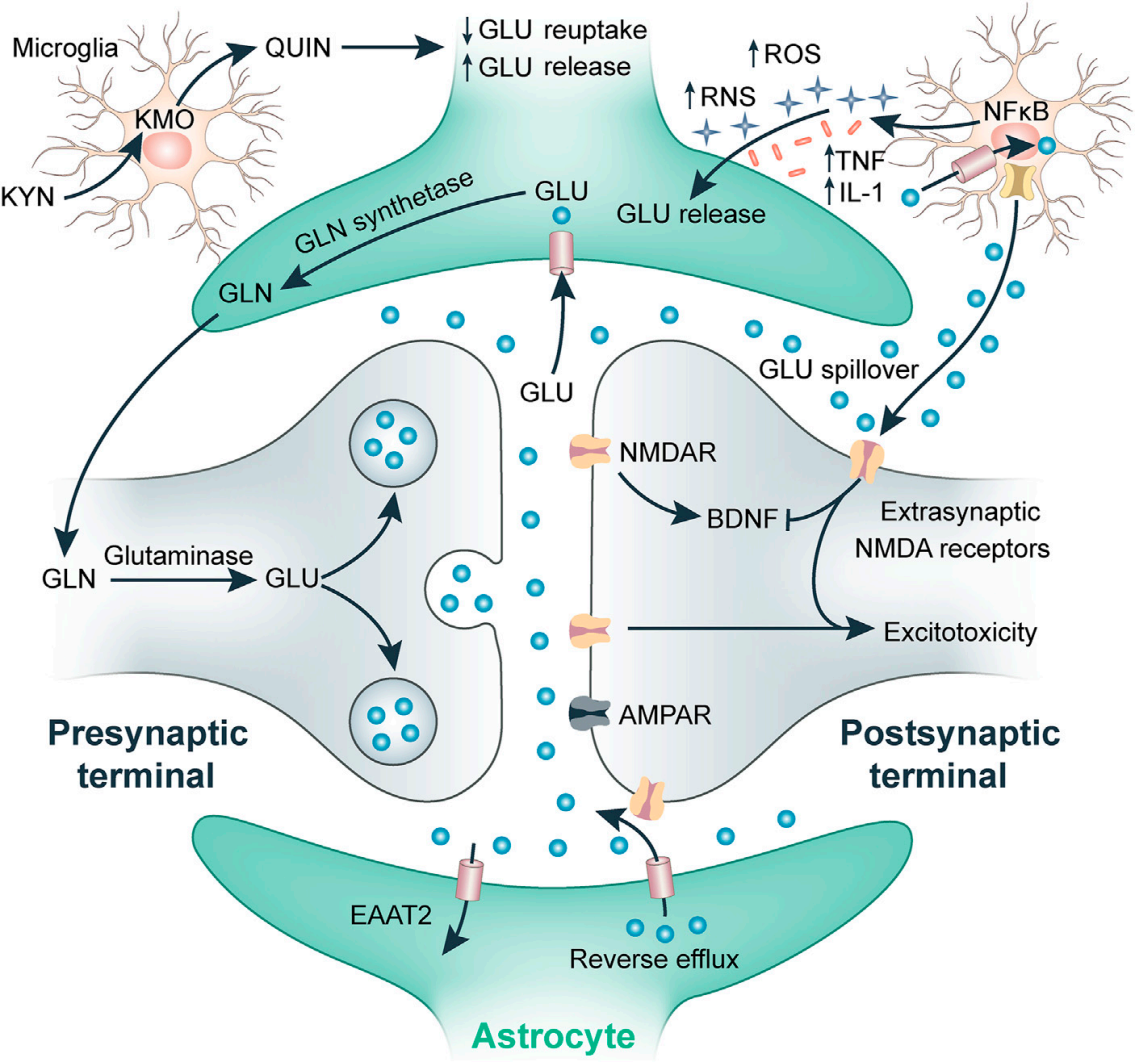
Effect of inflammation on glutamate in the synapse.

Glutamate metabolism imbalance and inflammatory immune responses may be the key mechanisms underlying the comorbidity of PTSD with CHD (O’Donnell et al., 2021). As an excitatory neurotransmitter, glutamate plays an important role in maintaining the excitability of the central nervous system; however, it is also a strong neurotoxin that functions in learning and memory behaviors (Simioni et al., 2018). Abnormal glutamate energy metabolism leads to stress responses and PTSD (Manning and Toker, 2017). Glutamate plays an important role in PTSD and is closely related to the formation of memory, specifically long-term memory, during the occurrence of PTSD, suggesting that glutamate is an important risk factor for learning and memory impairments (Averill et al., 2016). The role of glutamate in PTSD is partially mediated by regulation of the hypothalamic-pituitary-adrenal (HPA) axis. Animal studies have shown that overexpression of glutamate receptors reduces the release of adrenocorticotropic hormone in response to stress, which is essential for the initiation and maintenance of the HPA axis. Furthermore, enhanced neurotransmitter function of glutamate can promote the body to produce new memories, thereby reducing memories related to traumatic events (Kelmendi et al., 2016). When re-experiencing trauma, an individual with PTSD is unable to maintain adequate glutamate delivery because of an impaired glutamate system, which leads to increased levels of over-attention, stress response, and fear (Slagsvold et al., 2014). Pro-inflammatory cytokines, such as IL-1, IL-1β, IL-6, tumor necrosis factor alpha (TNFα), C-reactive protein (CRP), and interferon-γ, activate the HPA axis, promote excitatory glutamate, and damage the neuroplasticity of the brain by reducing the levels of neurotransmitters such as serotonin (5-HT), norepinephrine (NE), and dopamine (DA), which ultimately affects cognitive, behavioral, and emotional responses (Figure 2) (Manning and Toker, 2017; Simioni et al., 2018). Pro-inflammatory cytokines such as IL-1β, IL-6, TNFα, CRP, and interferon-γ activate the HPA axis response by reducing monoamine neurotransmitter levels in the central nervous system, thereby promoting glutamate excitotoxicity, damaging the plasticity of brain nerves, and ultimately affecting cognition, behavior, and emotional responses (Krugers et al., 2010; Grajeda-Iglesias and Aviram, 2018). Microglia are abnormally activated when the body receives stress or danger signals, resulting in the release of inflammatory cytokines, such as TNFα and IL-1β, and an excessive increase in glutamate release. These pro-inflammatory mediators re-activate astrocytes, leading to their release of inflammatory factors and further inducing abnormal activation of microglia, resulting in neuronal damage (Krugers et al., 2010).

AS is a chronic inflammatory disease characterized by lipid accumulation, smooth muscle cell proliferation, cell apoptosis, necrosis, fibrosis, and local inflammation. High glutamate levels are associated with the incidence of CHD (Vaarhorst et al., 2014). Furthermore, glutamine is linked to clinical manifestations of AS through an association with an increased risk of both plaque development and increased intima-media thickness (Würtz et al., 2012). A recent study reported contrary findings; in postmenopausal women, glutamine was the only metabolite associated with a decreased risk of CHD, whereas glutamate remained a biomarker after adjustment for conventional CHD risk factors (Paynter et al., 2018). Studies on the effects of all 20 amino acids on atherogenesis using murine macrophages identified six specific amino acids, including glutamate and glutamine, which significantly affected lipid accumulation in arterial cells at non-toxic level (Rom et al., 2017). A major protective effect on macrophage triglyceride metabolism was also observed, as shown by decreased uptake of triglyceride-rich very-low-density lipoprotein and macrophage triglyceride biosynthesis rate. Glutamate is characterized as a pro-atherogenic compound because it stimulates triglyceride accumulation in macrophages through upregulation of triglyceride biosynthesis. This is mediated by inducing key regulators of cellular triglyceride biosynthetic pathways, including sterol regulatory element-binding protein 1 (Horton et al., 2002) and diacylglycerol acyltransferase-1, which catalyzes the final step of this pathway (Yen et al., 2008). Additionally, glutamate exerts marked stimulatory effects on macrophage oxidative stress and overexpression of scavenger receptor class B type 1, a regulator of macrophage oxidative status and lipid metabolism (Ji et al., 2011; Liu et al., 2016). Inflammation also plays an important role in the occurrence and development of heart disease. The release of inflammatory factors leads to an increase in glutamate concentration, which further induces excitotoxicity *via* excessive Ca^2+^ influx and oxidative stress. Chronic inflammation is a precursor of myocardial infarction and ischemic stroke (Chen et al., 2016; Yang et al., 2017).

Glutamate affects the immune response by regulating the HPA axis to affect memory and learning, ultimately influencing the incidence of PTSD, as well as patient outcomes. At the same time, glutamate could increase inflammatory response and oxidative stress response, then promote plaque development and disrupt triglyceride metabolism, leading to the development of AS (Miao et al., 2020). Microglia are abnormally activated when the body receives stress or danger signals, resulting in the release of inflammatory cytokines, which in turn could increase glutamate concentration, further inducing excitotoxicity *via* excessive Ca^2+^ influx and oxidative stress. The bidirectional relationships among nervous system, systemic inflammation, and metabolic deterioration may affect the risks of PTSD and AS (Kaplan et al., 2018).

## 5 Effects of lipids on post-traumatic stress disorder comorbidity with atherosclerosis

A crucial step in early AS development is the infiltration of monocytes from the circulation into the arterial wall (Xu et al., 2015), where they differentiate into macrophages and accumulate lipids in a process known as macrophage foam cell formation, the hallmark feature of early atherogenesis (Dickhout et al., 2008). The accumulation of lipids, notably cholesterol and triglycerides, in macrophages, their conversion into foam cells, and the initiation and progression of atherosclerotic lesions are primarily determined by the balance between lipoprotein uptake by macrophages, lipid biosynthesis rate within macrophages, and lipid clearance from macrophages, known as cholesterol efflux (Libby et al., 2016; Rom and Aviram, 2016). Hyperlipidemic status may cause oxidized low-density lipoprotein (LDL) accumulation as the first step in the progression of AS. Macrophages play an important role in the inflammatory response, and after activation, they are involved in other immune cells in advanced atherosclerotic lesions. Cholesterol, triglyceride, and lipoprotein levels have been implicated in the pathogenesis of AS. Increased serum LDL and triglyceride level are responsible for the formation of atherosclerotic lesions (Albertini et al., 2002). Lipid metabolism and LDL modification are important in AS development. Lipid metabolism occurs *via* both exogenous and endogenous pathways. Retention of LDL particles in the vessel wall is considered the first step in AS pathogenesis (Wisniewska et al., 2017). Under smoking, hypertensive, hyperglycemic, and hyperlipidemic conditions, the production of reactive oxygen species (ROS) increases, overwhelming the endogenous antioxidant response. Lastly, oxidative stress increases LDL oxidation and impairs endothelial function (Frostegård et al., 2003; Bloomer, 2007; Zhou et al., 2013). Similarly, an excessive inflammatory response is a major cause of the formation, development, and rupture of atherosclerotic plaques (Bentzon et al., 2014). Key findings from two studies revealed a correlation between AS and several cytokines and chemokines, including TNF-α, IL-6, IL-1, IL-2, IL-7, IL-8, IL-10, IL-18, soluble tumor necrosis factor receptor, and CRP, which reflects the chronic low-grade systemic inflammation in AS (Zhoa and Mallat, 2019; Gencer et al., 2021). Inflammatory responses are believed to occur in all stages of AS. Increasing evidence suggests a bidirectional relationship between metabolic abnormalities and systemic inflammatory responses.

Lipids play important roles in the brain, including neurogenesis, synaptogenesis, myelin information, and impulse transduction (Cermenati et al., 2015). The availability of cholesterol is one of the limiting factors of synaptogenesis and is critical to its persistence. It is also important for the stability of neurotransmitters (Liu et al., 2010). The pathophysiology of PTSD includes synaptic loss (Krystal et al., 2017), increased myelination (Chao et al., 2015), abnormal white matter (Li et al., 2016), and reduced cortical thickness (Wrocklage et al., 2017), suggesting a role of lipids in the pathogenesis of PTSD. Sanacora et al. (2022) found that metabolic abnormalities play a critical role in mediating the psychopathological effects of stress. Abnormal lipid metabolism and hemodynamic changes can lead to increased production of ROS, accumulation of inflammatory substances, and induction of systemic inflammation, resulting in cognitive impairment (Jha et al., 2017). In case of excessive lipid accumulation in neurons and astrocytes, inflammatory factors such as vascular cell adhesion molecule increase, blood–brain barrier permeability increases, hippocampal neurogenesis and synapse numbers decrease, hippocampal-dependent spatial memory and other cognitive abilities decline, and anxiety-like behaviors increase (Ji et al., 2011). Pro-inflammatory cytokines, such as IL-1β, IL-6, TNFα, CRP, and interferon-γ activate neuroendocrine responses (including the HPA axis) and promote glutamate excitatory toxicity by reducing the levels of monoaminergic neurotransmitters (such as 5-HT, NE, and DA) in the central nervous system. Damage to brain neuroplasticity and other mechanisms ultimately affects cognitive, behavioral, and emotional responses (Sanacora et al., 2022). A recent systematic review suggested dysregulation of lipids that may serve as biomarker to predict the risk of PTSD (Bharti et al., 2022).

Hyperlipidemic status may cause oxidized LDL accumulation. When lipid accumulation in neurons and astrocytes is excessive, inflammatory factors such as vascular cell adhesion molecule and blood–brain barrier permeability increase, hippocampal neurogenesis and synapse numbers decrease, hippocampal-dependent spatial memory and other cognitive abilities decline, and anxiety-like behaviors increase. AS may lead to increased inflammatory responses in the brain, abnormal microvessels, reduced synaptic plasticity, and cognitive impairment (Baker et al., 2018; Asim et al., 2021). Current clinical and basic studies indicate that metabolic abnormalities and immune-inflammatory responses play an important role in the comorbidity of PTSD and AS.

## 6 Effects of lipid peroxidation and ferroptosis comorbidity with atherosclerosis

Ferroptosis is an iron-dependent oxidative form of cell death associated with increased lipid peroxidation and insufficient capacity to eliminate lipid peroxides (Galluzzi et al., 2018). Abnormal lipid metabolism, oxidative stress and inflammation are the main features of AS. Different signal pathways have demonstrated that ferroptosis, an iron-driven form of programmed cell death characterized by lipid peroxidation, contributes to the onset and progression of AS (Wang et al., 2021). The main mechanism of ferroptosis is the Fenton reaction, which involves intracellular free iron interacting with hydrogen peroxide to deplete plasma membrane polyunsaturated fatty acids (PUFAs) (Stockwell et al., 2017). Numerous cellular metabolic processes, such as redox balance, iron management, mitochondrial activity, and the metabolism of amino acids, lipids, and carbohydrates, control ferroptosis. The sulfhydryl-dependent redox system and the mevalonate pathway are two metabolic mechanisms that influence cellular vulnerability to ferroptosis (Zheng and Conrad, 2020). Restricted GSH production, disruptions in iron homeostasis, an accumulation of lipid peroxides, and fatty acid synthesis are all factors that contribute to the development of ferroptosis and are also intimately related to AS (Wang et al., 2021). NRF2-Keap1 pathway decreases ferroptosis associated with AS by maintaining cellular iron homeostasis, increasing the production glutathione, GPX4 and NADPH (Dodson et al., 2019). The p53 plays different roles in ferroptosis at different stages of AS in a transcription-dependent and transcription independent manner (Tarangelo et al., 2018). p53 targets gene GLS2 (glutaminases2), relating to glutaminolysis, also involved in ferroptosis (Gao et al., 2015). Glutaminolysis (a major source of anaplerosis) is involved in ferroptosis through ferroptosis functioning of the tricarboxylic acid (TCA) cycle. Importantly, loss of fumarate hydrase function, a TCA cycle component and tumor suppressor, confers resistance to cysteine-deprivation induced ferroptosis (Gao et al., 2019). The activation of ferroptosis has been shown to be a factor in the progression of AS through the Hippo pathway. AS and ferroptosis are caused by additional transcription factors such ATF3, ATF4, and STAT3. A few proteins or enzymes are also involved in the regulation of ferroptosis and AS (Wang et al., 2019).

## 7 AMPK/mTOR pathway in post-traumatic stress disorder comorbidity with atherosclerosis

**FIGURE 3:**
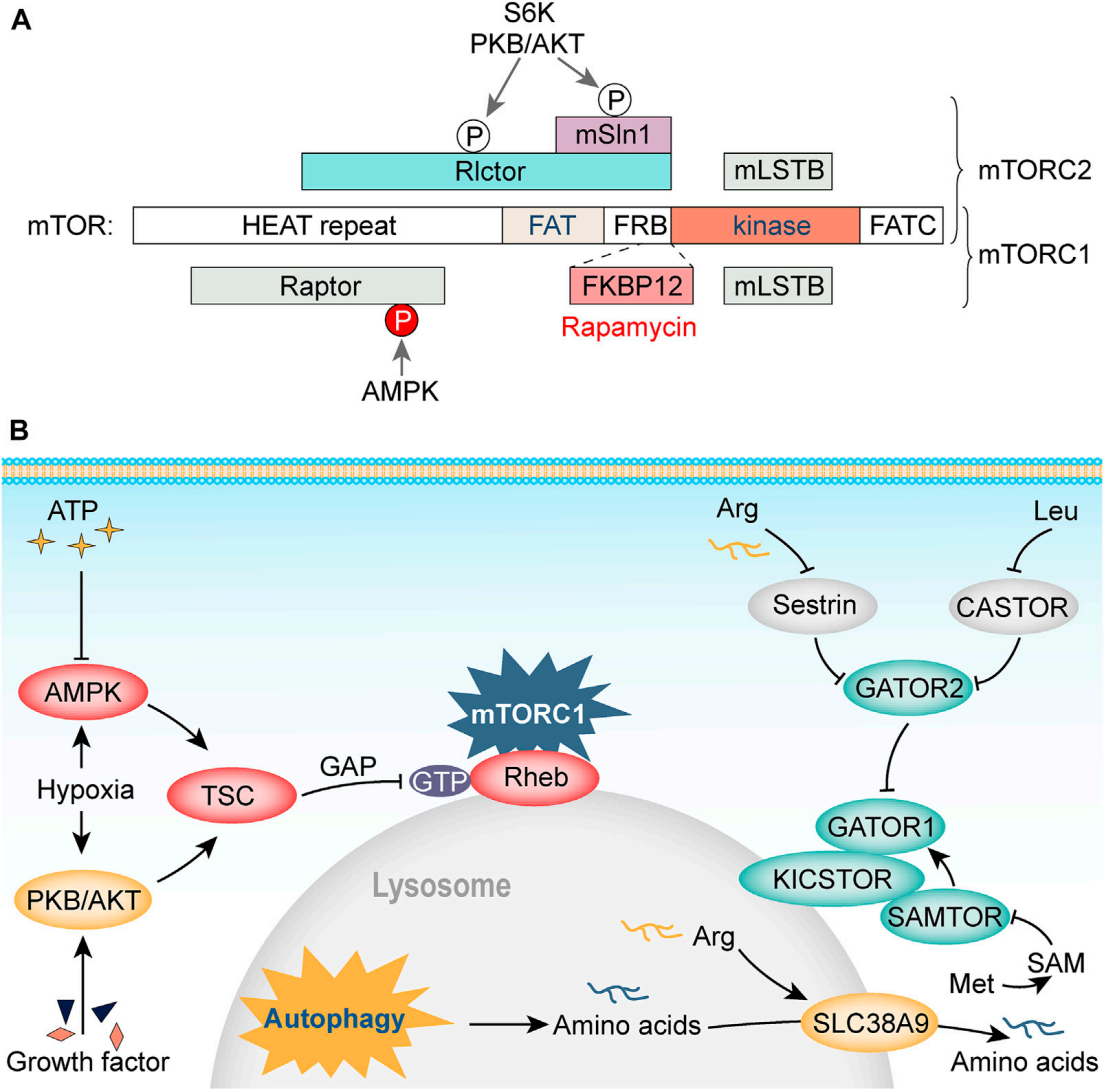
**(A, B)** AMPK/mTOR metabolic signaling module.

Although PTSD and AS are speculated to share a common pathway in metabolic imbalance and the immune inflammatory response (O’Donnell et al., 2021), in-depth mechanistic studies are still needed. A large number of molecular mechanism studies have revealed the relationship among energy metabolism, synaptic plasticity, and the related signaling pathways. Among these, the AMPK/mTOR energy metabolism-related pathway is an attractive area of research in the fields of metabolism, immunity, and autophagy. AMPK, a cellular energy sensor that is highly sensitive to the intracellular AMP/ATP ratio, is activated when this ratio increases to regulate glucose and lipid metabolism, and is related to autophagy (Lin and Hardie, 2018). AMPK activation promotes uncoordinated-51-like kinase 1 (ULK1) activity and decreases its mobility (Jia et al., 2019). Activated ULK1 interacts with AMPK to promote autophagy progression under stress conditions, including hunger and ischemia (Mao and Klionsky, 2011). Previous studies involving AMPK’s downstream target, mTOR, have demonstrated the key role of the AMPK/mTOR pathway in neurogenesis and synaptic plasticity (Fidaleo et al., 2017). Regulation of metabolism, proliferation, apoptosis, and autophagy by mTOR is directly activated by both serine/threonine kinase 11 (LKB1)-AMPK-mTOR pathway and phosphorylation of its downstream 4E-binding protein 1, eIF4E, and ribosomal S6 protein kinase (Cheon and Cho, 2021). The mammalian target of rapamycin complex 1 (mTORC1) regulates lipid synthesis by regulating protein synthesis, acting on sterol response element binding protein, and negatively regulating autophagy *via* the phosphorylation of ULK1, thus interfering with the connection between AMPK and ULK1 (Kim et al., 2013). mTORC2 primarily regulates cell proliferation and survival, cytoskeletal remodeling, cell migration, and glucose and amino acid metabolism (Figure 3) (Chun and Kim, 2021).

AMPK/mTOR plays an important role in the metabolic and inflammatory responses in cardiovascular diseases and psychiatric disorders. mTOR regulates the expression of inflammatory cytokines in LDL-induced macrophages (Ai et al., 2014) and interferes with lipid metabolism, leading to the development of AS (Ma et al., 2013). Zheng et al. (2016) used rapamycin to inhibit mTOR activity and reduce energy consumption in protein synthesis to maintain normal ATP levels while inhibiting the mitochondrial function of neurons. A previous animal study found that short-term exposure of hippocampal neurons to amyloid-β oligomers leads to a decrease in intracellular ATP levels and AMPK activity, resulting in a decrease in the number of glucose transporters GLUT3/4 on the dendritic surface of hippocampal neurons and in the ability of neurons to transport glucose (Da Silva et al., 2017).

## 8 PI3K/akt pathway in post-traumatic stress disorder comorbidity with atherosclerosis

PI3K is a lipid kinase that plays an active role in cell survival and energy metabolism. Akt is a serine/threonine protein kinase that regulates apoptosis, proliferation, and glucose metabolism (Simioni et al., 2018). The PI3K/Akt pathway is closely related to cardiac development, myocardial hypertrophy, and myocardial apoptosis regulation (Manning and Toker, 2017). Clinical studies have reported that overexpression of Akt after coronary artery bypass transplantation may help promote the survival of cardiac cells and recovery of cardiac function (Slagsvold et al., 2014). Animal studies have shown that Akt activation reduces the size of myocardial infarction area and significantly reduces myocardial cell apoptosis in a mouse model, and these effects can be blocked by an Akt pathway-specific inhibitor (LY294002) (Liu et al., 2016). Moreover, Akt activation inhibits oxidative stress injury in cardiomyocytes and hydrogen peroxide-induced apoptosis of cardiomyocytes in ischemia–reperfusion injury (Chen et al., 2016). Various factors simultaneously act on the mitochondrial membrane, resulting in the opening of mitochondrial permeability conversion hole and increased expression of pro-apoptotic proteins such as Bax, p21, and caspase. The cell then enters programmed death in oxidative stress injury. However, the activated PI3K/Akt pathway inhibits the interaction between apoptotic proteins and blocks the mitochondrial apoptosis pathway (Ma et al., 2013; Yang et al., 2017). Therefore, the PI3K/Akt pathway plays an important role in oxidative stress-induced apoptosis of cardiomyocytes.

Previous studies on PTSD have reported that fear, anxiety, and stress-related mood disorders are based on persistent abnormal neurobiological responses to traumatic events (Liu et al., 2018b; Kaplan et al., 2018). The Akt signaling pathway ameliorates PTSD symptoms by promoting synaptic plasticity and glutamate transmission (Liu et al., 2018b). Glutamate receptors are the main excitatory neurotransmitter receptors in the mammalian brain that determine synaptic transmission efficiency (Liu et al., 2018a; Barnes et al., 2020) and play an important role in learning and memory (Zarebidaki et al., 2020). Increasing evidence indicates that abnormal glutamate energy systems are associated with stress responses and PTSD (Ji et al., 2011). Patients with PTSD and impaired glutamate systems cannot maintain adequate glutamate delivery when re-experiencing trauma-related stimuli, leading to increased over-attention, stress response, and fear (Pitman et al., 2012). Activated PI3K/Akt is crucial for the formation of fear memory, and the PI3K/Akt pathway may underlie the anti-regression of fear memory caused by traumatic stress (Yang et al., 2015) and be related to the formation of anxiety-like symptoms in PTSD (Knox et al., 2021). Akt plays an important role in neural development, learning, and memory, and upregulation of the PI3K/Akt pathway may prevent and treat cognitive impairment (Palumbo et al., 2021; Tong et al., 2021). Chinese medicine and exercise have been shown to enhance the PI3K/Akt pathway, regulate synaptic plasticity, resist apoptosis and inflammation, promote the regression of fear memory, increase 5-HT levels in the hippocampus, and alleviate PTSD symptoms (Ling et al., 2020; Zhang et al., 2020).

## 9 Conclusion

Existing literature supports an important role of several metabolites, including glutamate and lipids, in the pathogenesis of PTSD and AS. Glutamate may affect the immune response and oxidative stress response, ultimately influencing the incidence of PTSD and AS, as well as patient outcomes. Abnormal lipid metabolism leads to the increased production of ROS, accumulation of inflammatory substances, and induction of a systemic inflammatory response, resulting in cognitive dysfunction and plaque development. AMPK/mTOR and PI3K-Akt energy metabolism-related pathways have attracted considerable attention in studies on metabolism and inflammation. These pathways may affect PTSD and AS by mediating metabolic pathways and inflammation to interfere with metabolism and immunity. However, how AMPK/mTOR and PI3K/Akt regulate metabolism and inflammation in the interaction between PTSD and AS requires further clarification. Future studies are necessary to explore the role of the AMPK/mTOR and PI3K/Akt pathways in PTSD and AS using molecular biology techniques. The expected results will provide new ideas for the precise diagnosis, treatment, and intervention of PTSD comorbidity with AS, which has important social significance and great economic value for the development of targeted drugs, exploration of efficient prevention and control strategies, and reduction of the disease burden.

## References

[B1] Ahmed H. M. Blaha M. J. Nasi r. K. Rivera J. J. Blumenthal R. S. (2012). Effects of physical activity on cardiovascular disease. Am. J. Cardiol. 109, 288–295. 10.1016/j.amjcard.2011.08.042 22011559

[B2] Ai D. Jiang H. Westerterp M. Westerterp M. Murphy A. J. Wang M. (2014). Disruption of mammalian target of rapamycin complex 1 in macrophages decreases chemokine gene expression and atherosclerosis. Circ. Res. 114, 1576–1584. 10.1161/CIRCRESAHA.114.302313 24687132 PMC4058053

[B3] Albertini R. Moratti R. De Luca G. (2002). Oxidation of low-density lipoprotein in atherosclerosis from basic biochemistry to clinical studies. Curr. Mol. Med. 2, 579–592. 10.2174/1566524023362177 12243250

[B4] Asim M. Wang B. Hao B. Wang X. G. (2021). Ketamine for post-traumatic stress disorders and it's possible therapeutic mechanism. Neurochem. Int. 146, 105044. 10.1016/j.neuint.2021.105044 33862176

[B5] Averill L. A. Purohit P. Averill C. L. Boesl M. A. Krystal J. H. Abdallah C. G. (2016). Glutamate dysregulation and glutamatergic therapeutics for PTSD: Evidence from human studies. Neurosci. Lett. 649, 147–155. 10.1016/j.neulet.2016.11.064 27916636 PMC5482215

[B6] Baker S. K. Chen Z. L. Strickland S. Revenko A. S. MacLeod A. R. (2018). Blood-derived plasminogen drives brain inflammation and plaque deposition in a mouse model of Alzheimer's disease. Proc. Natl. Acad. Sci. USA. 115, E9687–E9696. 10.1073/pnas.1811172115 30254165 PMC6187132

[B7] Barnes J. R. Mukherjee B. Rogers B. Nafar F. Gosse M. Parsons M. P. (2020). The relationship between glutamate dynamics and activity-dependent synaptic plasticity. J. Neurosci. 40, 2793–2807. 10.1523/JNEUROSCI.1655-19.2020 32102922 PMC7117894

[B8] Bentzon J. F. Otsuka F. Virmani R. Falk E. (2014). Mechanisms of plaque formation and rupture. Circ. Res. 114, 1852–1866. 10.1161/CIRCRESAHA.114.302721 24902970

[B9] Bharti V. Bhardwaj A. Elias D. A. Metcalfe A. W. S. Kim J. S. (2022). A systematic review and meta-analysis of lipid signatures in post-traumatic stress disorder. Front. Psychiatry. 13, 847310. 10.3389/fpsyt.2022.847310 35599759 PMC9120430

[B10] Birchenough G. Schroeder B. O. Backhed F. Hansson G. C. (2019). Dietary destabilisation of the balance between the microbiota and the colonic mucus barrier. Gut Microbes 10, 246–250. 10.1080/19490976.2018.1513765 30252606 PMC6546334

[B11] Bloomer R. J. (2007). Decreased blood antioxidant capacity and increased lipid peroxidation in young cigarette smokers compared to nonsmokers: Impact of dietary intake. Nutr. J. 6, 39. 10.1186/1475-2891-6-39 17996062 PMC2174505

[B12] Bookwalter D. B. Roenfeldt K. A. LeardMann C. A. Kong S. Y. Riddle M. S. Rull R. P. (2020). Posttraumatic stress disorder and risk of selected autoimmune diseases among US military personnel. BMC psychiatry 20, 23–28. 10.1186/s12888-020-2432-9 31941473 PMC6964079

[B13] Cermenati G. Mitr o. N. Audano M. Melcangi R. C. Crestani M. De Fabiani E. (2015). Lipids in the nervous system: From biochemistry and molecular biology to patho-physiology. Biochim. Biophys. Acta 1851, 51–60. 10.1016/j.bbalip.2014.08.011 25150974

[B14] Chao L. L. Tosun D. Woodward S. H. Kaufer D. Neylan T. C. (2015). Preliminary evidence of increased hippocampal myelin content in veterans with posttraumatic stress disorder. Front. Behav. Neurosci. 9, 333–341. 10.3389/fnbeh.2015.00333 26696852 PMC4667092

[B15] Chen X. G. Lv Y. X. Zhao D. Zhang L. Zheng F. Yang J. Y. (2016). Vascular endothelial growth factor-C protects heart from ischemia/reperfusion injury by inhibiting cardiomyocyte apoptosis. Mol. Cell. Biochem. 413, 9–23. 10.1007/s11010-015-2622-9 26769665

[B16] Cheon S. Y. Cho K. J. (2021). Lipid metabolism, inflammation, and foam cell formation in health and metabolic disorders: Targeting mTORC1. J. Mol. Med. 99, 1497–1509. 10.1007/s00109-021-02117-8 34312684

[B17] Chun Y. Kim J. (2021). AMPK-mTOR signaling and cellular adaptations in hypoxia. Int. J. Mol. Sci. 22, 9765–9788. 10.3390/ijms22189765 34575924 PMC8465282

[B18] Cryan J. F. O'Riordan K. J. Cowan C. S. M. Sandhu K. V. Bastiaanssen T. F. S. Boehme M. (2019). The microbiota-gut-brain axis. Physiol. Rev. 99, 1877–2013. 10.1152/physrev.00018.2018 31460832

[B19] Da Silva G. S. S. Melo H. M. Lourenco M. V. Silva N. M. L. E. de Carvalho M. B. Alves-Leon S. V. (2017). Amyloid-β oligomers transiently inhibit AMP-activated kinase and cause metabolic defects in hippocampal neurons. J. Biol. Chem. 292, 7395–7406. 10.1074/jbc.M116.753525 28302722 PMC5418041

[B20] Dickhout J. G. Basseri S. Austin R. C. (2008). Macrophage function and its impact on atherosclerotic lesion composition, progression, and stability: The good, the bad, and the ugly. Arterioscler. Thromb. Vasc. Biol. 28, 1413–1415. 10.1161/ATVBAHA.108.169144 18650503

[B21] Dodson M. Castro-Portuguez R. Zhang D. D. (2019). NRF2 plays a critical role in mitigating lipid peroxidation and ferroptosis. Redox Biol. 23, 101107. 10.1016/j.redox.2019.101107 30692038 PMC6859567

[B22] Ebrahimi R. Lynch K. E. Beckham J. C. Dennis P. A. Viernes B. Tseng C. H. (2021). Association of posttraumatic stress disorder and incident ischemic heart disease in women veterans. JAMA Cardiol. 6, 642–651. 10.1001/jamacardio.2021.0227 33729463 PMC7970390

[B23] Edmondson D. von Knel R. (2017). Post-traumatic stress disorder and cardiovascular disease. Lancet Psychiatry 4, 320–329. 10.1016/S2215-0366(16)30377-7 28109646 PMC5499153

[B24] Fidaleo M. Cavallucci V. Pani G. (2017). Nutrients, neurogenesis and brain ageing: From disease mechanisms to therapeutic opportunities. Biochem. Pharmacol. 141, 63–76. 10.1016/j.bcp.2017.05.016 28539263

[B25] Fossion P. Leys C. Kempenaers C. Braun S. Verbanck P. Linkowski P. (2015). Beware of multiple traumas in PTSD assessment: The role of reactivation mechanism in intrusive and hyper-arousal symptoms. Aging & Ment. health 19, 258–263. 10.1080/13607863.2014.924901 24927132

[B26] Foster J. A. Rinaman L. Cryan J. F. (2017). Stress & the gut-brain axis: Regulation by the microbiome. Neurobiol. stress 7, 124–136. 10.1016/j.ynstr.2017.03.001 29276734 PMC5736941

[B27] Frostegård J. Ruihua W. U. Lemne C. Thulin T. Witztum J. L. De Faire U. (2003). Circulating oxidized low-density lipoprotein is increased in hypertension. Clin. Sci. 105, 615–620. 10.1042/CS20030152 12837127

[B28] Gajeda-Iglesias C. Aviram M. (2018). Specific amino acids affect cardiovascular diseases and atherogenesis via protection against macrophage foam cell formation: Review article. Rambam Maimonides Med. J. 9, e0022. 10.5041/RMMJ.10337 29944113 PMC6115485

[B29] Galluzzi L. Vitale I. Aaronson S. A. Abrams J. M. Adam D. Agostinis P. (2018). Molecular mechanisms of cell death: Recommendations of the nomenclature committee on cell death 2018. Cell. Death Differ. 25, 486–541. 10.1038/s41418-017-0012-4 29362479 PMC5864239

[B30] Gao M. Monian P. Quadri N. Ramasamy R. Jiang X. (2015). Glutaminolysis and transferrin regulate ferroptosis. Mol. Cell. 59, 298–308. 10.1016/j.molcel.2015.06.011 26166707 PMC4506736

[B31] Gao M. Yi J. Zhu J. Minikes A. M. Monian P. Thompson C. B. (2019). Role of mitochondria in ferroptosis. Mol. Cell. 73, 354–363. 10.1016/j.molcel.2018.10.042 30581146 PMC6338496

[B32] Gencer S. Evans B. R. van der Vorst E. P,C. Doring Y. Weber C. (2021). Inflammatory chemokines in atherosclerosis. Cells 10, 226–252. 10.3390/cells10020226 33503867 PMC7911854

[B33] Hemmings S. M. J. Malan-Muller S. van den Heuvel L. L. Demmitt B. A. Stanislawski M. A. Smith D. G. (2017). The microbiome in posttraumatic stress disorder and trauma-exposed controls: An exploratory study. Psychosom. Med. 79, 936–946. 10.1097/PSY.0000000000000512 28700459 PMC5763914

[B34] Horton J. D. Goldstein J. L. Brown M. S. (2002). SREBPs: Activators of the complete program of cholesterol and fatty acid synthesis in the liver. J. Clin. Invest. 109, 1125–1131. 10.1172/JCI15593 11994399 PMC150968

[B35] Jha S. K. Jha N. K. Kumar D. Ambasta R. K. Kumar P. (2017). Linking mitochondrial dysfunction, metabolic syndrome and stress signaling in Neurodegeneration. Biochimica Biophysica Acta (BBA)-Molecular Basis Dis. 1863, 1132–1146. 10.1016/j.bbadis.2016.06.015 27345267

[B36] Ji A. Meyer J. M. Cai L. Akinmusire A. de Beer M. C. Webb N. R. (2011). Scavenger receptor SR-BI in macrophage lipid metabolism. Atherosclerosis 217, 106–112. 10.1016/j.atherosclerosis.2011.03.017 21481393 PMC3139003

[B37] Jia J. Abudu Y. P. Claude-Taupin A. Gu Y. X. Kumar S. Choi S. W. (2019). Galectins control MTOR and AMPK in response to lysosomal damage to induce autophagy. Autophagy 15, 169–171. 10.1080/15548627.2018.1505155 30081722 PMC6287685

[B38] Jie Z. Xia H. Zhong S.-L. Feng Q. Li S. Liang S. (2017). The gut microbiome in atherosclerotic cardiovascular disease. Nat. Commun. 8, 845–912. 10.1038/s41467-017-00900-1 29018189 PMC5635030

[B39] Kaplan G. B. Leite-Morris K. A. Wang L. Rumbika K. K. Heinrichs S. C. Zeng X. (2018). Pathophysiological bases of comorbidity: Traumatic brain injury and post-traumatic stress disorder. J. Neurotrauma. 35, 210–225. 10.1089/neu.2016.4953 29017388

[B40] Karlsson F. H. Fak F. Nookaew I. Tremaroli V. Fagerberg B. Petranovic D. (2012). Symptomatic atherosclerosis is associated with an altered gut metagenome. Nat. Commun. 3, 1245–1248. 10.1038/ncomms2266 23212374 PMC3538954

[B41] Kelmendi B. Adams T. G. Yarnell S. Southwick S. Abdallah C. G. Krystal J. H. (2016). Ptsd: From neurobiology to pharmacological treatments. Eur. J. Psychotraumatol. 7, 31858. 10.3402/ejpt.v7.31858 27837583 PMC5106865

[B42] Kim J. Kim Y. C. Fang C. Russell R. C. Kim J. H. Fan W. (2013). Differential regulation of distinct Vps34 complexes by AMPK in nutrient stress and autophagy. Cell. 152, 290–303. 10.1016/j.cell.2012.12.016 23332761 PMC3587159

[B43] Knox D. Della Valle R. Mohammadmirzaei N. Shultz B. Biddle M. Farkash A. (2021). PI3K-Akt signaling in the basolateral amygdala facilitates traumatic stress enhancements in fear memory. Int. J. Neuropsychopharmacol. 24, 229–238. 10.1093/ijnp/pyaa083 33151288 PMC7968623

[B44] Krugers H. J. Hoogenraad C. C. Groc L. (2010). Stress hormones and AMPA receptor trafficking in synaptic plasticity and memory. Nat. Rev. Neurosci. 11, 675–681. 10.1038/nrn2913 20820185

[B45] Krystal J. H. Abdallah C. G. Averill L. A. Kelmendi B. Harpaz-Rotem I. Sanacora G. (2017). Synaptic loss and the pathophysiology of PTSD: Implications for ketamine as a prototype novel therapeutic. Curr. Psychiatry Rep. 19, 74–85. 10.1007/s11920-017-0829-z 28844076 PMC5904792

[B46] Laudani S. Torrisi S. A. Alboni S. Bastiaanssen T. F. S. Benatti C. Rivi V. (2023). Gut microbiota alterations promote traumatic stress susceptibility associated with p-cresol-induced dopaminergic dysfunctions. Brain, Behav. Immun. 107, 385–396. 10.1016/j.bbi.2022.11.004 36400332

[B47] Li L. Sun G. Liu K. Li M. Li B. Qian S. W. (2016). White matter changes in posttraumatic stress disorder following mild traumatic brain injury: A prospective longitudinal diffusion tensor imaging study. Chin. Med. J. 129, 1091–1099. 10.4103/0366-6999.180518 27098796 PMC4852678

[B48] Libby P. Bornfeldt K. E. Tall A. R. (2016). Atherosclerosis: Successes, surprises, and future challenges. Circ. Res. 118, 531–534. 10.1161/CIRCRESAHA.116.308334 26892955 PMC4762065

[B49] Lin S. C. Hardie D. G. (2018). Ampk: Sensing glucose as well as cellular energy status. Cell. Metab. 27, 299–313. 10.1016/j.cmet.2017.10.009 29153408

[B50] Ling L. Jie C. Han Z. W. Yun N. W. Li L. W. Dan Y. (2020). Study on mechanism of *Guilu Erxianjiao* in treatment of post-traumatic stress disorder based on network pharmacology. Zhongguo Zhong Yao Za Zhi 45, 1816–1823. 10.19540/j.cnki.cjcmm.20190929.401 32489065

[B51] Liu J. P. Tang Y. Zhou S. Toh B. H. McLean C. Li H. (2010). Cholesterol involvement in the pathogenesis of neurodegenerative diseases. Mol. Cell. Neurosci. 43, 33–42. 10.1016/j.mcn.2009.07.013 19660552

[B52] Liu S. S. Ai Q. D. Feng K. Li Y. B. Liu X. (2016). The cardioprotective effect of dihydromyricetin prevents ischemia-reperfusion-induced apoptosis *in vivo* and *in vitro* via the PI3K/Akt and HIF-1 alpha signaling pathways. Apoptosis 21, 1366–1385. 10.1007/s10495-016-1306-6 27738772

[B53] Liu G. H. Feng D. Y. Wang J. Zhang H. F. Peng Z. W. Cai M. (2018a). rTMS ameliorates PTSD symptoms in rats by enhancing glutamate transmission and synaptic plasticity in the ACC via the PTEN/Akt signalling pathway. Mol. Neurobiol. 55, 3946–3958. 10.1007/s12035-017-0602-7 28550530

[B54] Liu R. Tang A. L. Wang X. Y. Chen X. Zhao L. Xiao Z. M. (2018b). Inhibition of lncRNA NEAT1 suppresses the inflammatory response in IBD by modulating the intestinal epithelial barrier and by exosome-mediated polarization of macrophages. Int. J. Mol. Med. 42, 2903–2913. 10.3892/ijmm.2018.3829 30132508

[B55] Ma K. L. Liu J. Wang C. X. Ni J. Zhang Y. Wu Y. (2013). Increased mTORC1 activity contributes to atherosclerosis in apolipoprotein E knockout mice and in vascular smooth muscle cells. Int. J. Cardiol. 168, 5450–5453. 10.1016/j.ijcard.2013.03.152 23972959 PMC3827505

[B56] Malan-Muller S. Valles-Colomer M. Foxx C. L. Vieira-Silva S. van den Heuvel L. L. Raes J. (2022). Exploring the relationship between the gut microbiome and mental health outcomes in a posttraumatic stress disorder cohort relative to trauma-exposed controls. Eur. Neuropsychopharmacol. 56, 24–38. 10.1016/j.euroneuro.2021.11.009 34923209

[B57] Manning B. D. Toker A. (2017). AKT/PKB signaling: Navigating the network. Cell. 169, 381–405. 10.1016/j.cell.2017.04.001 28431241 PMC5546324

[B58] Mao K. Klionsky D. J. (2011). AMPK activates autophagy by phosphorylating ULK1. Circ. Res. 108, 787–788. 10.1161/RES.0b013e3182194c29 21454792 PMC3619191

[B59] Miao J. Zang X. Cui X. Zhang J. (2020). Autophagy, hyperlipidemia, and atherosclerosis. Autophagy Biol. Dis. 1207, 237–264. 10.1007/978-981-15-4272-5_18 32671753

[B60] Musazzi L. Tornese P. Sala N. Popoli M. (2018). What acute stress protocols can tell us about PTSD and stress-related neuropsychiatric disorders. Front. Pharmacol. 9, 758. 10.3389/fphar.2018.00758 30050444 PMC6052084

[B61] O'Donnell C. J. Schwartz L. L. Cohen B. E. Fayad Z. A. Gillespie C. F. Liberzon I. (2021). Posttraumatic stress disorder and cardiovascular disease: State of the science, knowledge gaps, and research opportunities. JAMA Cardiol. 6, 1207–1216. 10.1001/jamacardio.2021.2530 34259831

[B62] Palumbo S. Paterson C. Yang F. Hood V. L. Law A. J. (2021). PKBβ/AKT2 deficiency impacts brain mTOR signaling, prefrontal cortical physiology, hippocampal plasticity and select murine behaviors. Mol. Psychiatry. 26, 411–428. 10.1038/s41380-020-00964-4 33328589 PMC7854513

[B63] Paone P. Cani P. D. (2020). Mucus barrier, mucins and gut microbiota: The expected slimy partners? Gut 69, 2232–2243. 10.1136/gutjnl-2020-322260 32917747 PMC7677487

[B64] Paynter N. P. Balasubramanian R. Giulianini F. Wang D. D. Tinker L. F. Gopal S. (2018). Metabolic predictors of incident coronary heart disease in women. Circulation 137, 841–853. 10.1161/CIRCULATIONAHA.117.029468 29459470 PMC5854187

[B65] Phan L. Chen L. D. Iacobucci M. Ho R. Majeed A. McIntyre R. S. (2020). Impact of COVID-19 pandemic on mental health in the general population: A systematic review. J. Affect. Disord. 277, 55–64. 10.1016/j.jad.2020.08.001 32799105 PMC7413844

[B66] Pitman R. K. Rasmusson A. M. Koenen K. C. Shin L. M. Orr S. P. Gilbertson M. W. (2012). Biological studies of post-traumatic stress disorder. Nat. Rev. Neurosci. 13, 769–787. 10.1038/nrn3339 23047775 PMC4951157

[B67] Rom O. Aviram M. (2016). Endogenous or exogenous antioxidants vs. pro-oxidants in macrophage atherogenicity. Curr. Opin. Lipidol. 27, 204–206. 10.1097/MOL.0000000000000287 26959710

[B68] Rom O. Grajeda-Iglesias C. Najjar M. Abu-Saleh N. Volkova N. Dar D. E. (2017). Atherogenicity of amino acids in the lipid-laden macrophage model system *in vitro* and in atherosclerotic mice: A key role for triglyceride metabolism. J. Nutr. Biochem. 45, 24–38. 10.1016/j.jnutbio.2017.02.023 28431321

[B69] Rossetti H. C. Weiner M. Hynan L. S. Cullum C. M. Khera A. Lacritz L. H. (2015). Subclinical atherosclerosis and subsequent cognitive function. Atherosclerosis 241, 36–41. 10.1016/j.atherosclerosis.2015.04.813 25957568 PMC4722814

[B70] Roy S. S. Foraker R. E. Girton R. A. Mansfield A. J. (2015). Posttraumatic stress disorder and incident heart failure among a community-based sample of US veterans. Am. J. Public Health 105, 757–763. 10.2105/AJPH.2014.302342 25713943 PMC4358172

[B71] Sanacora G. Yan Z. Popoli M. (2022). The stressed synapse 2.0: Pathophysiological mechanisms in stress-related neuropsychiatric disorders. Nat. Rev. Neurosci. 23, 86–103. 10.1038/s41583-021-00540-x 34893785

[B72] Simioni C. Martelli A. M. Zauli G. Vitale M. McCubrey J. A. Capitani S. (2018). Targeting the phosphatidylinositol 3-kinase/Akt/mechanistic target of rapamycin signaling pathway in B-lineage acute lymphoblastic leukemia: An update. J. Cell. Physiol. 233, 6440–6454. 10.1002/jcp.26539 29667769

[B73] Slagsvold K. H. Moreira J. B. N. Rognmo O. Høydal M. Bye A. Wisløff U. (2014). Remote ischemic preconditioning preserves mitochondrial function and activates pro-survival protein kinase Akt in the left ventricle during cardiac surgery: A randomized trial. Int. J. Cardiol. 177, 409–417. 10.1016/j.ijcard.2014.09.206 25456576

[B74] Song H. Fang F. Arnberg F. K. Mataix-Cols D. Fernández de la Cruz L. Almqvist C. (2019). Stress related disorders and risk of cardiovascular disease: Population based, sibling controlled cohort study. BMJ 365, I1255. 10.1136/bmj.l1255 PMC6457109 30971390

[B75] Stanislawski M. A. Frank D. N. Borengasser S. J. Ostendorf D. M. Ir D. Jambal P. (2021). The gut microbiota during a behavioral weight loss intervention. Nutrients 13, 3248. 10.3390/nu13093248 34579125 PMC8471894

[B76] Stockwell B. R. Angeli J. P. F. Bayir H. Bush A. I. Conrad M. Dixon S. J. (2017). Ferroptosis: A regulated cell death nexus linking metabolism, redox biology, and disease. Cell. 171, 273–285. 10.1016/j.cell.2017.09.021 28985560 PMC5685180

[B77] Tarangelo A. Magtanong L. Bieging-Rolett K. T. Li Y. Ye J. Attardi L. D. (2018). p53 suppresses metabolic stress-induced ferroptosis in cancer cells. Cell. Rep. 22, 569–575. 10.1016/j.celrep.2017.12.077 29346757 PMC5791910

[B78] Tong L. Li M. D. Nie P. Y. Chen Y. Chen Y. L. Ji L. L. (2021). miR-132 downregulation alleviates behavioral impairment of rats exposed to single prolonged stress, reduces the level of apoptosis in PFC, and upregulates the expression of MeCP2 and BDNF. Neurobiol. Stress. 14, 100311. 10.1016/j.ynstr.2021.100311 33718536 PMC7921013

[B79] Vaarhorst A. A. Verhoeven A. Weller C. M. Böhringer S. Göraler S. Meissner A. (2014). A metabolomic profile is associated with the risk of incident coronary heart disease. Am. Heart J. 168, 45–52. 10.1016/j.ahj.2014.01.019 24952859

[B80] Wang N. Zeng G.-Z. Yin J.-L. Bian Z.-X. (2019). Artesunate activates the ATF4-CHOP-CHAC1 pathway and affects ferroptosis in Burkitt's Lymphoma. Biochem. biophysical Res. Commun. 519, 533–539. 10.1016/j.bbrc.2019.09.023 31537387

[B81] Wang Y. Zhao Y. Ye T. Yang L. Shen Y. Li H. (2021). Ferroptosis signaling and regulators in atherosclerosis. Front. Cell Dev. Biol. 9, 809457. 10.3389/fcell.2021.809457 34977044 PMC8716792

[B82] Wilson M. A. Liberzon I. Lindsey M. L. Lokshina Y. Risbrough V. B. Sah R. (2019). Common pathways and communication between the brain and heart: Connecting post-traumatic stress disorder and heart failure. Stress 22, 530–547. 10.1080/10253890.2019.1621283 31161843 PMC6690762

[B83] Wisniewska A. Olszanecki R. Toton- Zuranska J. Kus K. Stachowicz A. Suski M. (2017). Anti-atherosclerotic action of agmatine in ApoE-knockout mice. Int. J. Mol. Sci. 18, 1706. 10.3390/ijms18081706 28777310 PMC5578096

[B84] Wrocklage K. M. Averill L. A. Scott J. C. Averill C. L. Schweinsburg B. Trejo M. (2017). Cortical thickness reduction in combat exposed U.S. veterans with and without PTSD. Eur. Neuropsychopharmacol. 27, 515–525. 10.1016/j.euroneuro.2017.02.010 28279623 PMC5429865

[B85] Würtz P. Raiko J. R. Magnussen C. G. Soininen P. Kangas A. J. Tynkkynen T. (2012). High throughput quantification of circulating metabolites improves prediction of subclinical atherosclerosis. Eur. Heart J. 33, 2307–2316. 10.1093/eurheartj/ehs020 22450427

[B86] Xu L. Dai P. X. Perrard J. L. Yang D. Xiao X. Teng B. B. (2015). Foamy monocytes form early and contribute to nascent atherosclerosis in mice with hypercholesterolemia. Arterioscler. Thromb. Vasc. Biol. 35, 1787–1797. 10.1161/ATVBAHA.115.305609 26112011 PMC4514542

[B87] Yang Z. Y. Quan H. Peng Z. L. Zhong Y. Tan Z. J. Gong Q. Y. (2015). Proton magnetic resonance spectroscopy revealed differences in the glutamate + glutamine/creatine ratio of the anterior cingulate cortex between healthy and pediatric post-traumatic stress disorder patients diagnosed after 2008 Wenchuan earthquake. Psychiatry Clin. Neurosci. 69, 782–790. 10.1111/pcn.12332 26171979

[B88] Yang K. Chen Z. X.’ Gao J. J. Shi W. N. Li L. F. Jiang S. (2017). The key roles of GSK-3 beta in regulating mitochondrial activity. Cell. Physiol. biochem. 44, 1445–1459. 10.1159/000485580 29190615

[B89] Yen C. L. Stone S. J. Koliwad S. Harris C. Farese R. V. (2008). Thematic review series: Glycerolipids. DGAT enzymes and triacylglycerol biosynthesis. J. Lipid Res. 49, 2283–2301. 10.1194/jlr.R800018-JLR200 18757836 PMC3837458

[B90] Zarebidaki F. Camacho M. Brockmann M. M. Trimbuch T. Herman M. A. Rosenmund C. (2020). Disentangling the roles of RIM and Munc13 in synaptic vesicle localization and neurotransmission. J. Neurosci. 40, 9372–9385. 10.1523/JNEUROSCI.1922-20.2020 33139401 PMC7724145

[B91] Zhang J. Xue R. Li Y. F. Zhang Y. Z. Wei H. W. (2020). Anxiolytic-like effects of treadmill exercise on an animal model of post-traumatic stress disorder and its mechanism. J. Sports Med. Phys. Fit. 60, 172–179. 10.23736/s0022-4707.20.10120-8 32008312

[B92] Zhao T. X. Mallat Z. (2019). Targeting the immune system in atherosclerosis: JACC state-of-the-art review. J. Am. Coll. Cardiol. 73, 1691–1706. 10.1016/j.jacc.2018.12.083 30947923

[B93] Zheng J. Conrad M. (2020). The metabolic underpinnings of ferroptosis. Cell. metab. 32, 920–937. 10.1016/j.cmet.2020.10.011 33217331

[B94] Zheng X. D. Boyer L. Jin M. J. Kim Y. Fan W. W. Bardy C. (2016). Alleviation of neuronal energy deficiency by mTOR inhibition as a treatment for mitochondria-related neurodegeneration. Elife 5, e13378–e13401. 10.7554/eLife.13378 27008180 PMC4846388

[B95] Zhou M. S. Chadipiralla K. Mendez A. J. Jaimes E. A. Silverstein R. L. Webster K. (2013). Nicotine potentiates proatherogenic effects of OxLDL by stimulating and upregulating macrophage CD36 signaling. Am. J. Physiol. Heart Circ. Physiol. 305, 563–574. 10.1152/ajpheart.00042.2013 PMC3891251 23748423

[B96] Ziganshina E. E. Sharifullina D. M. Lozhkin A. P. Khayrullin R. N. Ignatyev I. M. Ziganshin A. M. (2016). Bacterial communities associated with atherosclerotic plaques from Russian individuals with atherosclerosis. PloS one 11, e0164836. 10.1371/journal.pone.0164836 27736997 PMC5063344

